# Differential autophagic effects of vital dyes in retinal pigment epithelial ARPE-19 and photoreceptor 661W cells

**DOI:** 10.1371/journal.pone.0174736

**Published:** 2017-03-30

**Authors:** Shwu-Jiuan Sheu, Jiunn-Liang Chen, Youn-Shen Bee, Yi-An Chen, Shi-Han Lin, Chih-Wen Shu

**Affiliations:** 1 Department of Ophthalmology, Kaohsiung Veterans General Hospital, Kaohsiung, Taiwan; 2 School of Medicine, National Yang-Ming University, Taipei, Taiwan; 3 Department of Optometry, Chung Hwa University of Medical Technology, Tainan, Taiwan; 4 Yuh-Ing Junior College of Health Care & Management, Kaohsiung, Taiwan; 5 National Defense Medical Center, Taipei, Taiwan; 6 Department of Medical Education and Research, Kaohsiung Veterans General Hospital, Kaohsiung, Taiwan; Univerzitet u Beogradu, SERBIA

## Abstract

Indocyanine green (ICG) and brilliant blue G (BBG) are commonly used vital dyes to remove internal limiting membrane (ILM) in vitreoretinal surgery. The vital dyes have shown cytotoxic effects in ocular cells. Autophagy is a stress responsive pathway for either protecting cells or promoting cell death. However, the role of autophagy in ocular cells in response to the vital dyes remains unknown. In this study, we found that ICG and BBG reduced cell viability in both human retinal pigment epithelial ARPE-19 and mouse photoreceptor 661W cells. ICG and BBG induced lipidated GFP-LC3-II and LC3-II in ARPE-19 and 661W cells. Combination treatment with the autophagy inhibitor chloroquine indicated that ICG and BBG reduced autophagic flux in ARPE-19 cells, whereas the vital dyes induced autophagic flux in 661W cells. Moreover, genetic and pharmacological ablation of autophagy enhanced vital dyes-induced cytotoxicity in ocular cells. Dietary supplements, including resveratrol, lutein, and CoQ10, induced autophagy and diminished the cytotoxic effects of ICG and BBG in ocular cells. These results suggest that autophagy may protect ARPE-19 and 661W cells from vital dyes-induced damage.

## Introduction

For the past decade, the removal of the internal limiting membrane (ILM) has been an important step for anatomical and functional success in macular hole, macular pucker, and even retinal detachment surgeries [1–4]. Because of its anatomical characteristics, the ILM is challenging to identify during surgical procedures. With the assistance of a vital dye such as indocyanine green (ICG) or brilliant blue G (BBG), the technique is much easier. Therefore, the use of dyes to identify structures during vitreoretinal surgery, “chromovitrectomy,” has become a popular technique in recent years [5]. Although the dyes are used temporarily during the operation, some of the dyes may remain on the unpeeled part of the ILM. Several groups have reported that ICG may persist in the ocular cavity up to 6 weeks after its application during surgery [6, 7]. Several groups reported toxicity to retinal pigment epithelial cells [8] and the neurosensory retina, as well as cases of optic nerve atrophy, after the use of ICG [4, 9–12]. Therefore, several alternative dyes have been introduced for use in vitreoretinal surgery, including infracyanine green (IfCG), trypan blue (TB), bromophenol bue (BPH), patent blue [8], and BBG.

Even so, all of the previously mentioned dyes were reported to exhibit toxicity on RPE cells following acute exposure during surgical doses [13]. IfCG, BBG, and BPH have been shown to be less toxic on retinal ganglion cells and RPE cells compared with ICG [14]. BBG, it was claimed, provided a good staining to the ILM and was not toxic in experimental studies and a case series in humans [15]. However, recent reports showed a selective toxicity to photoreceptors related to BBG after intravitreal injection in rabbit eyes and RPE changes on fluorescein angiography, as well as macular damage following accidental subretinal dye injection in humans [16–20]. Another report of the intraocular safety of ICG, TB, Evans blue (EB), and BBG on ARPE-19 cell lines and murine retinal ganglion/Müller glial (RGC) primary cell cultures showed that all dyes demonstrated relatively safe viability profiles in both cell lines at surgically relevant concentrations and times. BBG was the only dye that caused toxicity in ARPE-19 cell lines after short exposure times, and ICG had a favorable viability profile at almost all of the concentrations and times tested [21]. Mitochondria have been implicated in the cytotoxicity caused by the dyes. Mitochondrial membrane potential (ΔΨ_m_) was altered after exposure to surgical does of ICG, TB, PB, or a four-fold surgical dose of BrB [13]. An *in vitro* RPE cell study by Penha *et al*. revealed that expression of Bax, cytochrome c, and caspase-9 was upregulated at the mRNA and protein level after ICG exposure, but Bcl-2, an anti-apoptotic protein, was downregulated; however, brilliant blue (BriB), resulted in upregulation of Bcl-2 [11]. During real-life procedures, not only the RPE, but also the photoreceptors may be exposed to the dyes cause cytotoxicity in the these cells. Until now, no study had focused on the effect of the intraoperative dyes on the *in vitro* safety of photoreceptor cells. Cultures of 661W cells, an *in vitro* model that mimics photoreceptor cells, have been widely used in the study of retinal degeneration, retinal neuroprotection, and retinal regeneration [22].

Macroautophagy is typically referred to as a degradation process that proceeds in a lysosome-dependent manner by which microtubule-associated proteins 1A/1B light chain 3B (LC3) facilitates elongatation of autophagosome and fuses with lysosomes for degradation and recycling. Sequestome 1 (SQSTM1) contains LC3 and ubiquitin-binding motifs to recruit ubiquitinated proteins to the autophagosome, which serves as an autophagy receptor, for selective bulk degradation [23]. Autophagy plays a beneficial role in several ocular cell types to maintain the eye’s normal physiological function [24]. Autophagy is involved in maintaining inner segment turnover in photoreceptors, and it protects cells from stress and melanin degradation in RPE cells [24]. However, autophagy is activated to promote autophagic cell death in retinal ganglion cells during chronic intraocular pressure elevation, suggesting the role of autophagy might be varied depending on types of ocular cells or stress [25]. The role of autophagy in RPE and photoreceptor cells in response to vital dyes remains unknown. Here, we examine the autophagic effects of vital dyes in RPE and photoreceptor cells and showed that autophagy was inhibited and induced in ARPE-19 and 661W cells, respectively, when exposed to ICG and BBG. Nevertheless, ablation of autophagy enhanced ICG and BBG-induced cytotoxicity in both ARPE-19 and 661W cells. Administration of dietary supplements, including resveratrol, lutein, and CoQ10, induced autophagy and diminished the cytotoxic effects of ICG and BBG, suggesting autophagy may play a cytoprotective role in RPE and photoreceptor cells during exposure to ICG or BBG.

## Material and methods

### Compounds

The dyes ICG (I2633) and BBG (B0770) and the dietary supplements resveratrol (R5010), coenzyme Q10 (CoQ10, C9538), and lutein (xanthophyll from marigold, X6205) were purchased from Sigma-Aldrich (St. Louis, MO, USA). Reagents and materials for cell culture were obtained from GIBCO (Life Technologies; City, Country). CellTiter-Glo Luminescent Cell Viability Assay (G7572) was purchased from Promega Corporation (Madison, WI, USA).

### Dye preparation

ICG and BBG (5mg each) was dissolved in 100 μL dimethyl sulfoxide (DMSO) and phosphate-buffered saline (PBS) was added to obtain a stock of 5 mg/mL. The ICG solution should be made fresh before use. ICG and BBG were mainly used at concentrations of 0.05 mg/mL in this study. The surgical concentrations of ICG and BBG solutions are 0.5 mg/mL and 0.25 mg/mL, respectively.

### Cell culture of human RPE cells

Adult human RPE cell cultures (ARPE-19) were obtained from the American Type Culture Collection (CRL-2302; ATCC, Manassas, VA, USA). These cells were maintained in a 1:1 mixture of Dulbecco's modified Eagle's medium (DMEM) and Ham's F12 medium supplemented with 10% fetal bovine serum (Life Technologies), sodium bicarbonate (1.2 g/L), L-glutamine (2.5 mM), HEPES (15 mM), and sodium pyruvate (0.5 mM). The cells were cultured at 37°C in a humidified atmosphere of 95% air and 5% CO_2_. The mouse photoreceptor–derived 661W cell line was generously provided by Dr. Muayyad Al-Ubaidi (University of Oklahoma, Norman, OK, USA). Cells were grown in DMEM supplemented with 10% fetal bovine serum in 100-mm tissue culture dishes in which cells typically were seeded at a concentration between 1 × 10^5^ and 1 × 10^6^ in 10 mL of growth medium. They were allowed to grow to 80%–90% confluence before harvesting for use in experiments.

### CellTiter-Glo luminescent cell viability assay

ARPE19 and 661W cells were plated at 3000–5000 cells/well in 96-well plates and incubated at 37°C in an incubator containing 5% CO_2_. Cell viability was assessed using the CellTiter-Glo luminescent cell viability kit (G7572) from Promega Corporation according to the manufacturer’s instructions. The method is based on quantitation of the adenosine triphosphate present in the cells, detected by the generation of A luminescent signal. All experiments were repeated at least three times.

### Autophagic flux measurement and immunoblotting

For more precise monitoring of autophagic activity, cells were treated with or without 20 μM chloroquine (CQ; Sigma-Aldrich, C6628), an inhibitor of autophagy, for 2 h prior to harvesting. The cell lysates were used to detect LC3-II accumulation by immunoblotting to verify autophagic flux [26]. For immunoblotting, the cells were briefly rinsed in PBS (02-023-1, Biological Industries, City, Country) and lysed with RIPA buffer (1% NP40 [MDBio, City, Country, 101-9016-45-9], 50 mM Tris HCl, pH 7.5, 150 mM NaCl, 0.25% sodium deoxycholate [Sigma-Aldrich, D6750], 0.1% sodium dodecyl sulfate [SDS; Calbiochem, City, Country, 428015], and protease inhibitor cocktail [Roche, City, Country, 11873580001]). The cell lysates were resolved by sodium dodecyl sulfate–polyacrylamide gel electrophoresis and transferred electrophoretically onto nitrocellulose membranes. The membrane was blocked with 5% skim milk and then incubated with primary antibodies against LC3 (L7543), and ACTB (β-actin, A5441) (all from Sigma-Aldrich), SQSTM1 (BD Pharmingen, 610832), ATG5 (8540) and ATG7 (8558) (all purchased from Cell Signaling Technology) overnight at 4°C. The proteins were probed with an HRP-labeled secondary antibody (Santa Cruz, sc-2004 or sc-2005) and detected with an ECL reagent. The membrane was scanned and analyzed for the protein expression level with the ChemiDoc XRS Imaging System (Bio-Rad, Hercules, CA, USA).

### GFP-LC3 punctuation

GFP-LC3 plasmids (1500 cells/40 μL) were purchased from Addgene and were transfected into human ARPE-19 cells or mouse 661W cells for stable selection with G418 (600 μg/mL). The stable cells were seeded with ICG or BBG in plates for 30 min and recovered for 6 h. The cells were then fixed with 3.7% paraformaldehyde and cell images were collected with a 40× objective for GFP-LC3 puncta. Next, GFP-LC3–labeled autophagosomes were detected with fluorescence spot numbers and areas in each cell after ICG or BBG treatment. The percentages of changes of GFP-LC3 were calculated. Alternatively, the cells expressing GFP-LC3 were exposed to vital dyes and trypsinized for cell harvest. The cells were washed with PBS once and then resuspended with 200 μL 0.05% saponin to remove nonlipidated GFP-LC3 from cells [27]. One mL PBS was added to the cells to quantificate membrane-bound GFP-LC3-II with flow cytometry as reported previously [27].

### Transfection and shRNA infection

For siRNA transfection, the cells were seeded at 20% to 30% confluence and reversely transfected with RNAiMAX (Life Technologies; 13778–150) in the presence of 10 nM scrambled siRNA (Life Technologies, 12935–112), siRNA against ATG5 (GE Healthcare Dharmacon, City, Country, 9474) or ATG7 (Life Technologies, s20652) or BECN1 (Life Technologies, 4392420) for 72 h. For shRNA infection, shRNAs against *ATG5* (TRCN0000151963, The RNAi Consortium, Taiwan), *ATG7* (TRCN0000007584, The RNAi Consortium, Taiwan), and pLKO.1 Scrambled shRNA (Addgene, 1864). The plasmids were transfected into HEK293FT cells with Lipofectamine 2000 (Life Technologies, 11668–027) for 2 days, and the supernatant were used to infect ARPE-19 cells. The infected ARPE-19 cells were selected with puromycin (1 μg/ml) for 10 days to obtain stable knockdowned cells. The cells were harvested for knockdown efficiency by immunoblotting.

### Statistical analysis

All the data are expressed as the mean ± standard error of the mean from at least 3 individual experiments. The statistical analysis was performed using a nonparametric 2-tailed Student’s *t* test with Prism 5.0 (Graph-Pad, La Jolla, CA, USA). *P* values <0.05 were considered significant (* indicates *P*<0.05, ** indicates *P*<0.01, and *** indicates *P*<0.001).

## Results

### ICG and BBG diminish cell viability and modulate autophagy in ARPE-19 and 661W cells

**Fig 1:**
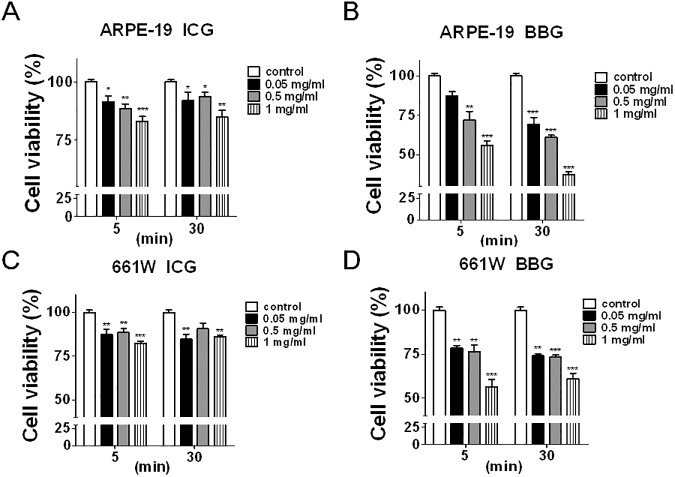
The effects of vital dyes on cell viability in ocular cells. (A and B) Human RPE cells ARPE-19 or (C and D) mouse photoreceptor cell 661W cells were treated with various concentration of ICG or BBG for 5 or 30 mins. The cells were then rinsed with PBS to remove the vital dyes and recover for 24 h. The cell viability of recovered cells was measured with Cell-titer Glo. The data were analyzed with Prism 5, and the results are shown as the means ± SEM from three independent experiments.

**Fig 2:**
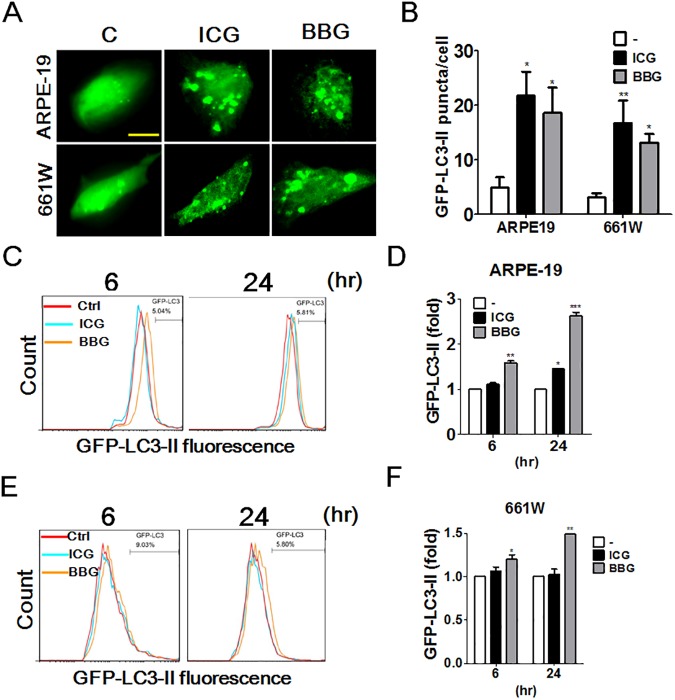
The effects of vital dyes on GFP-LC3 puncta and retention in treated-ARPE19 and 661W cells. ARPE19 and 661W cells harboring GFP-LC3 expression plasmid were treated with ICG or BBG (0.05 mg/ml) for 30 mins (A and B). Bar: 20 μm. The treated cells were recovered for 6 h and fixed to examine the GFP-LC3 puncta with fluorescence microscopy. ARPE19 (C and D) and 661W (E and F) cells expressing GFP-LC3 were treated with ICG or BBG (0.05 mg/ml) for 30 mins. The treated cells were recovered for 6 hr or 24 hr and fixed to examine the GFP-LC3 fluorescence intensity with flow cytometry. The quantitative results are shown as the means ± SEM from three independent experiments.

**Fig 3:**
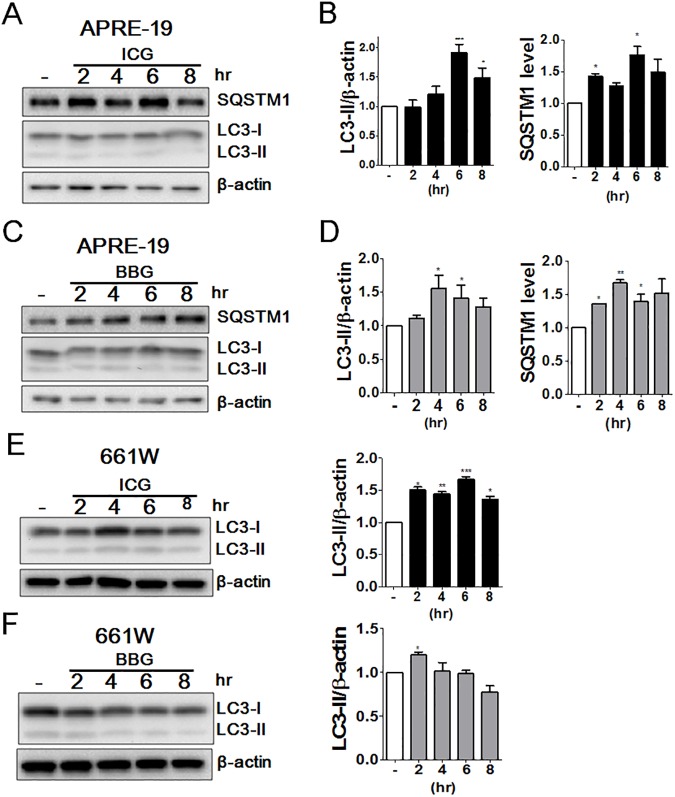
The effects of ICG and BBG on autophagy markers in ocular cells. Human RPE ARPE-19 cells were treated with (A and B) ICG (0.05 mg/ml) or (C and D) BBG (0.05 mg/ml) for 30 mins and then recovered with fresh media for different time period as indicated. The cells were harvested to determine the protein levels of autophagy markers SQSTM1 and LC3 with immunoblotting. Mouse photoreceptor cells 661W were treated with (E) ICG (0.05 mg/ml) or (F) BBG (0.05 mg/ml) for 30 mins and then recovered with fresh media for different time period as indicated. The cells were harvested to determine the protein levels of autophagy markers LC3 with immunoblotting. The quantitative results are shown as the means ± SEM from three independent experiments.

ARPE-19 and 661W cells were exposed to various concentrations (0.05, 0.5, or 1.0 mg/mL) of ICG or BBG for 5 or 30 min. The treated cells were then recovered for 24 h to determine the cytotoxic effects of ICG and BBG with CellTiter-Glo (Fig 1). Both ICG and BBG reduced cell viability in ARPE-19 cells (Fig 1A and 1B). In contrast with ICG, BBG treatment is relatively more cytotoxic to ocular cells. Because the cells treated with ICG and BBG at the dose of 0.05 mg/mL for 30 min reached the minimal cytotoxicity, these conditions were used in the subsequent experiments. Moreover, to corroborate whether ICG and BBG modulate autophagy in ocular cells, ARPE-19 and 661W cells expressing GFP-LC3 were exposed to ICG and BBG to examine the membrane-bound GFP-LC3 puncta (GFP-LC3-II) with confocal microscope (Fig 2A and 2B). ICG and BBG increased the GFP-LC3 puncta in both ARPE-19 and 661W cells. The GFP-LC3–expressing cells treated as above were also rinsed with the detergent saponin to remove nonlipidated GFP-LC3-I, and retained GFP-LC3-II, likely autophagosome associated form [27], were analyzed by flow cytometry (Fig 2C–2F). As with the results of fluorescent microscopy, the flow cytometry results showed that ICG and BBG increased lipidated GFP-LC3-II in ocular cells. Further, the cells treated with vital dyes were harvested to detect the level of the autophagy marker LC3 and adaptor SQSTM1 with immunoblotting (Fig 3). ICG increased the ratio of LC3-II/actin and the SQSTM1 level in ARPE-19 (Fig 3A–3D), suggesting ICG and BBG may inhibit autophagy in ARPE-19 according to the guideline of autophagy assays reported previously [8]. Although SQSTM1 was undetectable in 661W cells, the effects of vital dyes on lipidated LC3-II in 661W cells were similar to those in ARPE-19 (Fig 3E and 3F), suggesting that ICG and BBG modulate autophagy in ocular cells.

### ICG and BBG inhibited autophagic flux in ARPE-19 cells but induced autophagic flux in 661W cells

**Fig 4:**
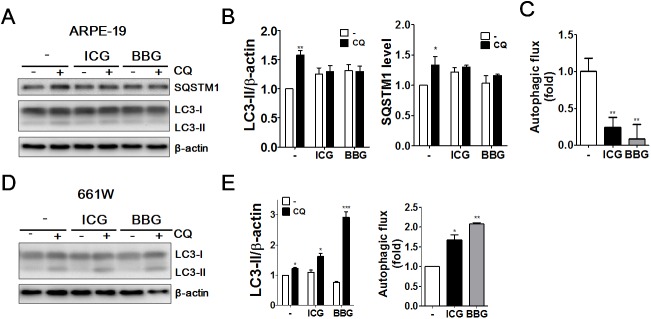
The effects of vital dyes on autophagic flux in ocular cells. Human RPE ARPE-19 cells were treated with ICG or BBG (0.05 mg/ml) for 30 mins in the presence or absence of autophagy inhibitor chloroquine (CQ, 20 μM) to determine the protein levels of autophagy markers with immunoblotting. The quantitative results for protein level of (B) SQSTM1、LC3-II/LC3-I conversion and (C) autophagic flux in treated cells were shown. (D) Photoreceptor cells 661W were treated with ICG or BBG (0.05 mg/ml) for 30 mins in the presence or absence of autophagy inhibitor chloroquine (CQ, 20 μM) to determine the protein levels of autophagy markers with immunoblotting. The quantitative results for LC3-II/LC3-I conversion and autophagic flux (E) in treated cells were shown. The quantitative results are shown as the means ± SEM from three independent experiments.

Autophagy induction transiently increases the GFP-LC3-II puncta and LC3-II/actin ratio in cells, whereas a block in autolysosomes causes an accumulation of GFP-LC3-II puncta and increases the LC3-II/actin ratio [26]. To precisely evaluate the autophagic effects of the vital dyes ICG and BBG in ARPE-19 cells and 661W cells, these cells were exposed to ICG and BBG in the presence or absence of the autophagy inhibitor CQ (Fig 4). The net difference in LC3-II levels between cells treated with and those without CQ was used to determine autophagic flux in the treated cells. Like the autophagy inhibitor CQ, ICG and BBG significantly increased the SQSTM1 protein level in ARPE-19 cells (Fig 4B). LC3-II flux was significantly decreased in ARPE-19 cells treated with ICG or BBG (Fig 4C). Conversely, ICG and BBG increased the LC3-II flux in photoreceptor 661W cells compared with the control cells (Fig 4D and 4E), indicating that vital dyes may suppress autophagy in retinal pigment epithelial ARPE-19 cells but induce autophagy in photoreceptor 661W cells. These results suggest vital dyes differentially modulate autophagy, which may depend on cell types.

### Autophagy protected ocular cells from ICG or BBG-induced damage

**Fig 5:**
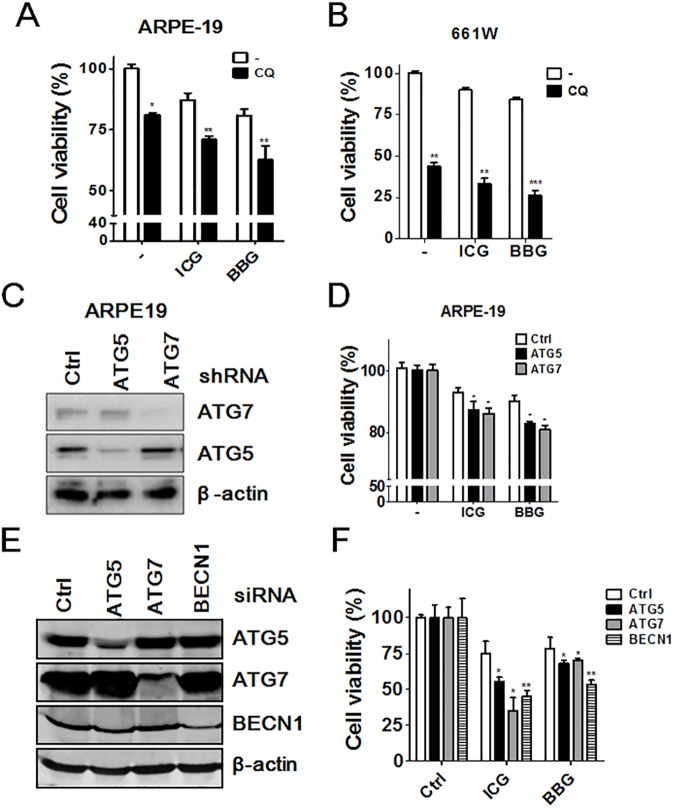
The effects of autophagy inhibition on cytotoxicity in ocular cells treated with vital dyes. (A) ARPE19 and (B) 661W cells were treated with autophagy inhibitor chloroquine (CQ, 20 μM) for 1 h. The cells were treated with ICG or BBG (0.05 mg/ml) for 30 mins and then recovered with CQ for 24 h to determine cell viability with CellTiter Glo. (C) ARPE-19 cells were infected with shRNA against ATG5 or ATG7 or (E) transfected with siRNA against ATG5, ATG7 or BECN1. The knockdown efficiency was verified with immunoblotting. (D and F) The knockdowned cells were exposed to ICG or BBG as panel A to evaluate cytotoxicity. The quantitative results are shown as the means ± SEM from three independent experiments.

**Fig 6:**
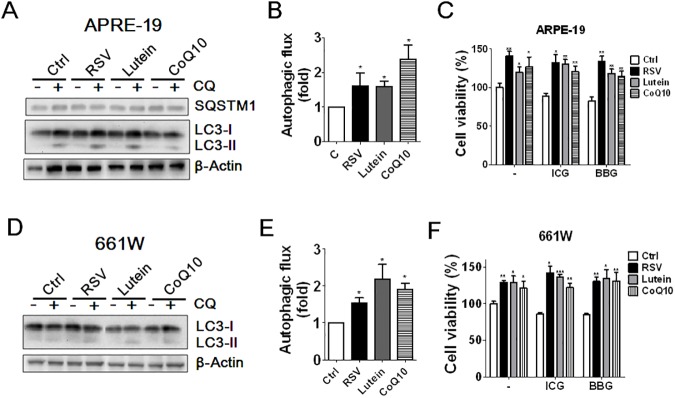
The effects of ocular supplements on autophagy and cell viability in vital dye-treated ocular cells. ***(***A)Human RPE ARPE-19 cells were treated with resveratrol (RSV, 10 μM), lutein (10 μM) or CoQ10 (10 μM) for 4 h in the presence or absence of autophagy inhibitor CQ (20 μM) to determine the protein level of LC3-II protein level with immunoblotting. (B) The net LC3-II between cells with and without CQ was used to quantitate LC3 flux. (C) ARPE-19 cells were exposed to ICG (0.05 mg/ml) or BBG (0.05 mg/ml) for 30 mins in the presence or absence of ocular supplements. The ICG and BBG were then removed from cells to recover for 24 h and examine the cell viability with CellTiter Glo. (D) Photoreceptor cells 661W were treated with resveratrol (RSV, 10 μM), lutein (10 μM) or CoQ10 (10 μM) for 4 h in the presence or absence of CQ (20 μM) to determine the protein levels of LC3-II and (E) quantitate autophagic flux. (F) 661W cells treated as panel C were used to examine the cell viability with CellTiter Glo. The quantitative results are shown as the means ± SEM from three independent experiments.

Autophagy can be protective or detrimental for cells in response to stresses. We examined the role of autophagy in ocular cells after treatment with ICG and BBG. Autophagy ablation with CQ notably enhanced the cytotoxicity of ICG and BBG in ARPE-19 and 661W cells (Fig 5A and 5B). Knockdown of *ATG5* or *ATG7 or BECN1*, which are essential genes for autophagy signaling, with shRNA or siRNA enhanced cytotoxic effects of ICG and BBG in ARPE-19 cells (Fig 5C–5F). Additionally, we examined the effect of three dietary supplements on autophagy in ocular cells, including resveratrol, lutein, and CoQ10. Among these dietary supplements, resveratrol has been reported to be an inhibitor of mechanistic target of rapamycin (mTOR) that induces autophagy [28]. As shown in Fig 6, resveratrol, lutein, and CoQ10 induced autophagic activity in both retinal pigment epithelial ARPE-19 cells and in photoreceptor 661W cells. Resveratrol and CoQ10 are reportedly to facilitate cell proliferation in neural progenitor cells and fibroblast, respectively [29, 30]. Indeed, pretreatment with the dietary supplements increased cell viability, likely due to promotion of cell proliferation as mentioned above. Furthermore, resveratrol, lutein, and CoQ10 diminished the cytotoxic effects of ICG and BBG in ocular cells compared to the untreated cells. These results suggest that autophagy may play a cytoprotective role in ocular cells in response to the damage of ICG and BBG.

## Discussion

Vital dyes have been reported to cause cytotoxicity in RPE cells, likely through mitochondrial damage and ROS production [13]. The role of autophagy in survival and death in ocular cells during stresses has been documented [31–33]. However, the involvement of autophagy in vital dyes–induced cytotoxicity in ocular cells remains unclear. Our study is the first to show that ICG and BBG induced cytotoxicity and modulated autophagy in both ARPE-19 and 661W cells. Interestingly, ICG and BBG inhibited autophagic flux in ARPE-19 cells, whereas the vital dyes induced autophagic flux in 661W cells. In addition, genetic and pharmacological ablation of autophagy diminished cell viability in both ARPE-19 and 661W cells. Dietary supplements, including resveratrol, lutein, and CoQ10, induced autophagy and diminished the cytotoxic effects of vital dyes in the ocular cells.

Autophagic response in the same stimuli might be varied in different cells. For example: doxorubicin induces autophagy in rat cardiac myocytes [34], whereas it reduces autophagy in mouse cardiac myocytes [35]. However, the mechanisms for differential autophagy regulation in difference cells remain unknown. Similarly, one of the interesting findings in our study is that ICG and BBG inhibited autophagic flux in ARPE-19 cells, whereas the vital dyes induced autophagic flux in 661W cells. Thus, the discrepancy in the responses to dyes between ARPE-19 and 661W cells may be through the different signaling pathways. In addition, ROS play a dual role on autophagy in various cells.

Autophagy is induced in cells in response to oxidative stress via several factors, such as Protein kinase RNA-like endoplasmic reticulum kinase (PERK), Hypoxia-inducible factor 1 (HIF1), p53, nuclear factor erythroid-2-related factor (NRF2), ATG4, and Forkhead box O3 (FOXO3) [36]. Conversely, ROS also down-regulates ULK1, a homologue of yeast ATG1, to inhibit autophagy via phosphorylation of p53 in NB4 cells [37]. We also noticed that ROS was induced by ICG and BBG in both ARPE-19 and 661W cells (S1 Fig). Nevertheless, elucidating these mechanisms requires further work to identify if ROS is involved in differential effects of vital dyes on autophagy in different ocular cells.

On the other hand, autophagy can reduce oxidative stress through the nuclear factor erythroid 2–related factor 2 (NRF2)/kelch-like ECH-associated protein 1 (KEAP1) and the SQSTM1 pathway [38, 39]. NRF2 is a transcription factor of the leucine zipper family and can activate antioxidant-defense genes such as glutathione peroxidase, superoxide dismutase, and thioredoxin [40]. Under normal conditions, NRF2 is sequestered by KEAP1 ubiquitination and degradation. Following oxidative stress, either the ubiquitin activity of KEAP1 is inhibited or SQSTM1 interacts with KEAP1 for its degradation, leading to the liberation and activation of NRF2, which in turn limits ROS production and cell damage. These results raise the possibility that the vital dyes may initially inhibit autophagy to elevate ROS and cause oxidative damage in ARPE-19 cells.

The RPE is a single layer of epithelial cells lining the posterior segment of the eye. It is located between the light-sensing photoreceptors and the choriocapillaris and serves essential function for vision. A failure of any one of these functions can lead to degeneration of the retina, loss of visual function, and blindness. Our results suggest that both ICG and BBG at the routine dose used in chromovitrectomy can affect autophagy not only RPE but also photoreceptor cells. Autophagy ablation with CQ or silencing ATG genes notably enhanced the cytotoxicity of ICG and BBG in ocular cells, indicating autophagy might be a survival mechanisms in ocular cells exposed to vital dyes. Moreover, Autophagy is a stress responsive pathway to limit damage-induced death, whereas autophagy may not be sufficient to block death in cells during excessive stresses. For example: autophagy is enhanced in cardiac cells during ischemia/reperfusion. Pretreatment with autophagy inducer rapamycin protects cells from ischemia/reperfusion injury [41]. Besides, many antioxidants are reportedly to promote cell proliferation and resistant to oxidative stress-induced cell death [29, 30], Three dietary supplements, which are reported to be antioxidants, induced autophagic activity and increased cell growth in both ARPE-19 cells and photoreceptor 661W cells. Pretreatment with the dietary supplements diminished the cytotoxic effects of ICG and BBG in ocular cells compared to the cells that did not receive the dietary supplements. Several mechanisms may be involved in cytoprotective effects of the dietary supplements in cells exposed to ICG or BBG, such as reduction of oxidative stress and promotion of mitochondrial biogenesis [42]. Our current results imply that dietary supplements-induced autophagy could be one of the mechanisms for protecting ocular cells from stress caused by ICG and BBG.

In conclusion, autophagy plays a protective role in ARPE-19 and 661W cells treated with vital dyes. The dietary supplements studied may protect the ocular cells against the vital dye–induced cytotoxicity via induction of autophagy.

## Supporting information

S1 Fig The effects of vital dyes on ROS production in ocular cells. (TIF)

## Abbreviations

ICG: Indocyanine green
BBG: brilliant blue G
ILM: internal limiting membrane
LC3: microtubule associated protein 1 light chain 3
SQSTM1: sequestosome 1
ATG: autophagy-related
BECN1: beclin 1
CQ: chloroquine
RPE: retinal pigment epithelial cells
ROS: reactive oxygen species
