# Supplementary figures and images for: Differential autophagic effects of vital dyes in retinal pigment epithelial ARPE-19 and photoreceptor 661W cells
Source: PLoS One. 2017 Mar 30;12(3):e0174736. doi: 10.1371/journal.pone.0174736 (PMC5373602; doi:10.1371/journal.pone.0174736)

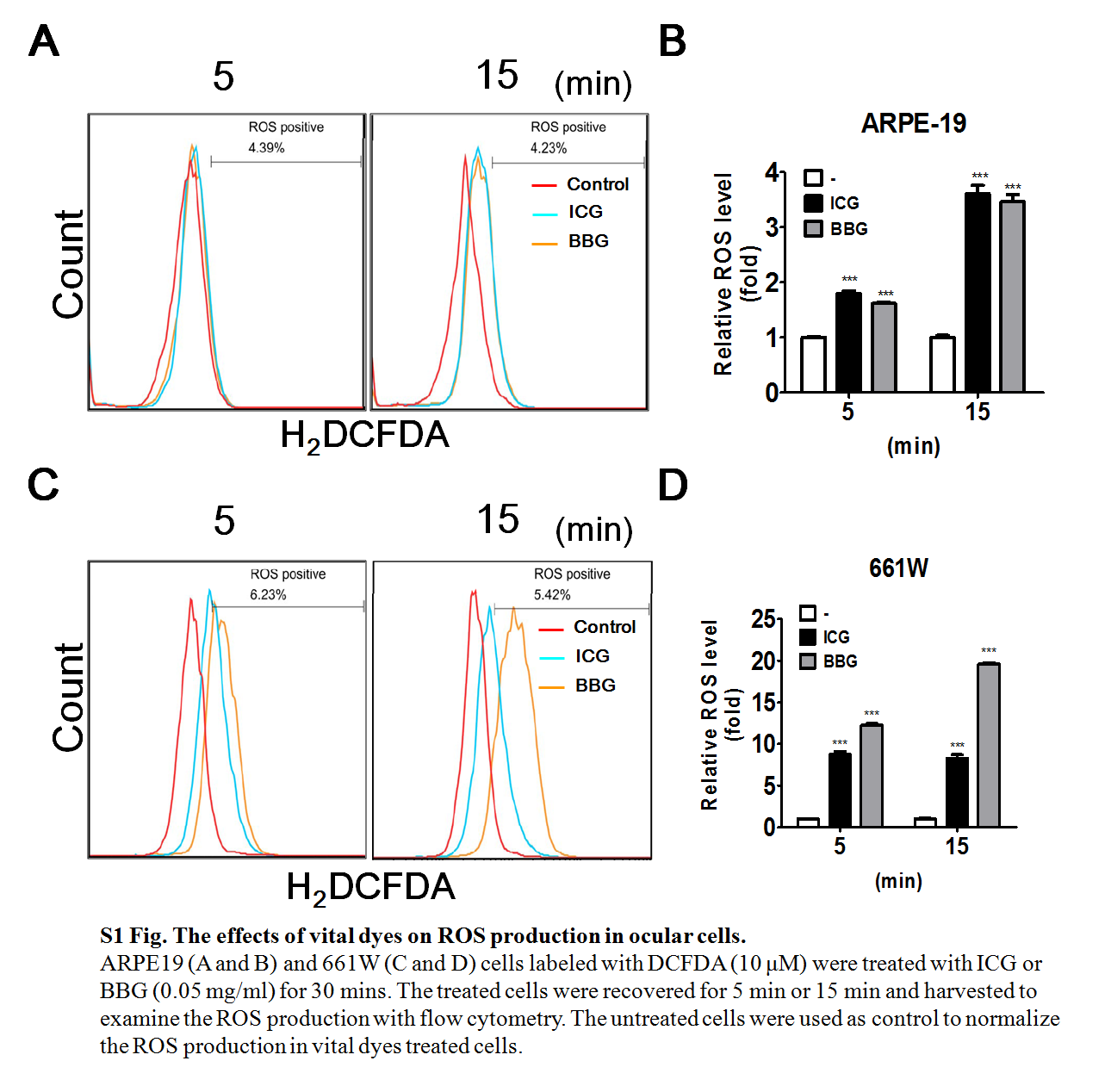

Supplement: S1 Fig — (TIF) [file pone.0174736.s001.tif]
